# Adenosine stimulates hepatic glycogenolysis via adrenal glands–liver crosstalk in mice

**DOI:** 10.1371/journal.pone.0209647

**Published:** 2018-12-21

**Authors:** Miki Tadaishi, Yutaro Toriba, Makoto Shimizu, Kazuo Kobayashi-Hattori

**Affiliations:** Department of Nutritional Science, Faculty of Applied Bioscience, Tokyo University of Agriculture, Tokyo, Japan; Universite Clermont Auvergne, FRANCE

## Abstract

Adenosine signaling is involved in glucose metabolism in hepatocytes and myocytes *in vitro*. However, no information is available regarding the effect of adenosine on glucose metabolism *in vivo*. Thus, we examined how extracellular adenosine acts on glucose metabolism using mice. Subcutaneous injections of adenosine (10, 25, and 50 mg/kg bodyweight) dose-dependently increased blood glucose levels, with the peak occurring at 30 min post injection. At 30 min after adenosine injection (25 mg/kg bodyweight), glycogen content in the liver, but not the skeletal muscle, was significantly decreased. Hepatic glycogen depletion by fasting for 12 h suppressed the increase of blood glucose levels at 30 min after adenosine injection. These results suggest that adenosine increases blood glucose levels by stimulating hepatic glycogenolysis. To investigate the effect of adenosine on the adrenal gland, we studied the glycogenolysis signal in adrenalectomized (ADX) mice. Adenosine significantly increased the blood glucose levels in sham mice but not in the ADX mice. The decrease in hepatic glycogen content induced by adenosine in the sham mice was partially suppressed in the ADX mice. The level of plasma corticosterone, the main glucocorticoid in mice, was significantly increased in the sham mice by adenosine but its levels were low in ADX mice injected with either PBS or adenosine. These results suggest that adenosine promotes secretion of corticosterone from the adrenal glands, which causes hepatic glycogenolysis and subsequently the elevation of blood glucose levels. Our findings are useful for clarifying the physiological functions of adenosine in glucose metabolism *in vivo*.

## Introduction

Blood glucose, which is used as the energy source for the entire body, is constantly regulated. During fasting, the liver is the main organ that produces glucose via glycogenolysis and gluconeogenesis to maintain a normal level in the blood. When the glycogen pool in the liver is exhausted, energy metabolism shifts from glucose to lipid metabolism [1].

Adenosine is an extracellular and intracellular molecule associated with energy metabolism [2], which is transported across cell membranes by nucleoside transporters. Physiological states involving the accumulation of extracellular adenosine include hypoxia [3] and exercise [4]. Extracellular adenosine is a signaling molecule that activates four different adenosine receptors (A1, A2a, A2b, and A3) which are categorized as G protein-coupled receptors. A1 and A3 inhibit adenylate cyclase activity via the Gi protein and decrease intracellular cyclic AMP (cAMP), a second messenger. In contrast, A2a and A2b stimulate adenylate cyclase activity via the Gs protein, which results in an increase in intracellular cAMP levels [5]. Adenosine receptors exist in various tissues, such as the liver, skeletal muscle, and adipose tissues [6], indicating that adenosine signaling controls many physiological reactions.

Adenosine receptors are associated with the regulation of energy metabolism in multiple tissues. For example, 8-cyclopentyl-1,3-dipropylxanthine, an A1 antagonist, modulates insulin-induced lipogenesis and epinephrine-induced lipolysis in isolated rat adipocytes [7]. A2b enhances cholesterol and triglyceride synthesis in the liver of ApoE knockout mice fed a high-fat diet [8]. These studies suggest that signaling through adenosine receptors is involved in lipid metabolism both *in vitro* and *in vivo*.

Adenosine receptors also participate in the regulation of glucose metabolism. Activation of A1 by the selective agonist N6-cyclopentyladenosine increases insulin-stimulated glucose uptake in isolated rat muscles [9]. Adenosine and 5′-N-ethylcarboxamidoadenosine (NECA), a non-selective agonist of adenosine receptors, stimulates glycogenolysis and gluconeogenesis in isolated rat hepatocytes [10]. In terms of glucose metabolism, there are several *in vitro* studies that show the effects of adenosine and adenosine receptors. However, the role of adenosine in glucose metabolism *in vivo* has not been elucidated.

In this study, we investigated the effect of adenosine on glucose metabolism in mice. Herein we report that adenosine affects glucose metabolism *in vivo* by stimulating hepatic glycogenolysis, partially through the secretion of corticosterone from adrenal glands.

## Materials and methods

### Experimental animals

Male 6-week-old C57BL/6J and C57BL/6N mice were obtained from CLEA Japan (Tokyo, Japan) and were housed in groups in cages at 23–25°C and 50%–60% humidity under a 12-h light/12-h dark cycle (lights on 08:00–20:00) with access to food and drinking water *ad libitum*. After acclimatization to a normal diet (MF, Oriental Yeast Co., Ltd., Tokyo, Japan) for 2 weeks, the mice were used in experiments. In the fasting condition to induce hepatic glycogen depletion, mice were starved for 12 h. In the adrenalectomized (ADX) mouse experiment, mice were ADX or sham operated on under pentobarbital anesthesia. Following all procedures, mice were observed in a warm environment until they are fully recovered from anesthesia. We observed the postoperative mice once daily for the 2 weeks and confirmed that there were no signs of pain, infection or dehiscence. The recovery period was 2 weeks and saline was given during this time.

The study was carried out in accordance with the recommendations in the Guide for the Care and Use of Laboratory Animals of the National Institutes of Health. All animal experiments were approved by the Animal Care and Research Ethics Committee of the Tokyo University of Agriculture (Permission No. 270127).

### Adenosine treatment

Mice were divided into groups based on glucose levels in whole blood collected from the tail vein using LIFE CHECK (EIDIA Co., Ltd., Tokyo, Japan). The mice were subcutaneously injected with adenosine (Wako Pure Chemicals Industries, Ltd., Osaka, Japan) dissolved in phosphate-buffered saline (PBS). The same volume of PBS was injected as a control. Post injection, glucose levels in whole blood were measured from the tail vein. The area under the curve (AUC) was calculated from glucose levels during the experiment.

### Plasma hormone analysis

For analysis of adrenocorticotropic hormone (ACTH), catecholamine, and corticosterone levels, whole blood was obtained from mice at post injection of 25 mg/kg bodyweight (bw) adenosine. Plasma samples were separated by centrifugation in the presence of EDTA, snap-frozen and stored at −80°C until analysis. ACTH, catecholamine, and corticosterone concentrations were measured with EIA kits (ACTH: Phoenix Pharmaceuticals, Inc., CA, USA; catecholamine: Abnova, Taipei City, Taiwan; and corticosterone: Yanaihara Institute Inc., Shizuoka, Japan). Plasma insulin levels were measured with mouse insulin sandwich ELISA kit (Shibayagi Co. Ltd., Gunma, Japan).

### Glycogen assay

The liver and quadriceps of mice were powdered under liquid nitrogen, and extracted in 0.3 M perchloric acid. Following the addition of 5 N hydrochloric acid, the suspensions were incubated at 100°C for 2 h. After neutralization with 5 N sodium hydroxide, the supernatant was recovered by centrifugation. The glycogen content in the supernatant was measured as glycosyl units [11] using the Glucose CII test kit (Wako Pure Chemicals Industries, Ltd.).

### Glucose-1-phosphate (G1P) assay

Quantification of hepatic G1P was performed by a colorimetric assay using a kit (BioVision Inc., CA, USA). Briefly, the liver were powdered under liquid nitrogen, and quantified according to the manufacturer’s instructions.

### Cholesterol assay

The adrenal glands were powdered under liquid nitrogen, then extracted in chloroform/methanol (2:1, v/v). After overnight incubation, the supernatant after centrifugation was dried under nitrogen and then 0.3 N potassium hydroxide solution in ethanol was added for the saponification reaction (incubated at 65°C for 20 min). The samples were re-extracted with chloroform/methanol and dried under nitrogen before dissolving in 10% triton X-100 in 2-propanol. The cholesterol levels of the samples were analyzed with a total cholesterol assay kit (Wako Pure Chemicals Industries, Ltd.).

### Histology of the adrenal gland

The adrenal glands were frozen in liquid nitrogen-cooled isopentane with OCT compound (Sakura Finetek Japan, Tokyo, Japan). After cutting 10 μm sections with a cryostat (Leica Microsystems GmbH, Wetzlar, Germany), lipid droplets were stained with Oil Red O (Sigma-Aldrich, St Louis, MO, USA) and nuclei were counterstained with hematoxylin (Muto Pure Chemicals Co., Ltd, Tokyo, Japan).

### Quantitative real-time RT-PCR

Whole adrenal glands were collected post injections with 0 and 25 mg/kg bw of adenosine, rapidly frozen in liquid nitrogen, and maintained at −80°C until use. Total RNA were prepared from the whole adrenal glands using ISOGENII (NIPPON GENE Co., LTD., Tokyo, Japan). cDNA was synthesized from total RNA using the PrimeScriptTM RT reagent kit with a gDNA eraser (Takara Bio Inc, Shiga, Japan). Quantitative real-time RT-PCR was performed using TUNDERBIRD SYBR qPCR Mix (TOYOBO Co., Ltd., Osaka, Japan), specific primers of target genes (Table 1), and Applied Biosystems 7300 (Thermo Fisher Scientific Inc., Waltham, MA, USA). Amplifications were performed under the following conditions: 1 min at 95°C, followed by 40 cycles of 15 s at 95°C and 1 min at 60°C. The mRNA level of target genes was normalized to that of the 36B4 gene.

**Table 1:** 

Target	Forward primer	Reverse primer
**StAR**	5′- GCTCTCTGCTTGGTTCTCAACTG -3′	5′-TTAGCACTTCGTCCCCGTTC-3′
**Cyp11b1**	5′-TCAGGCACAGTGTAGGGAAAAC-3′	5′-GCTGCAGTCGGTTGAAGTACC-3′
**SF-1**	5′-TTACTGGACAGGAGGTGGAGC-3′	5′-TACGAGGCTGTGGTTGTTCAG-3′
**36B4**	5′-GGCCCTGCACTCTCGCTTTC-3′	5′-TGCCAGGACGCGCTTGT-3′

### Statistical analysis

Statistical analysis was performed using SPSS 21.0 (SPSS, Inc., Chicago, IL, USA). Statistical differences were analyzed using a one-way ANOVA followed by Tukey’s multiple comparison test or Student’s t-test. Data are expressed as the mean ± standard error of the mean (SEM). All differences with P-values of *p* < 0.05 were considered statistically significant.

## Results

### Effect of adenosine on blood glucose levels

**Fig 1:**
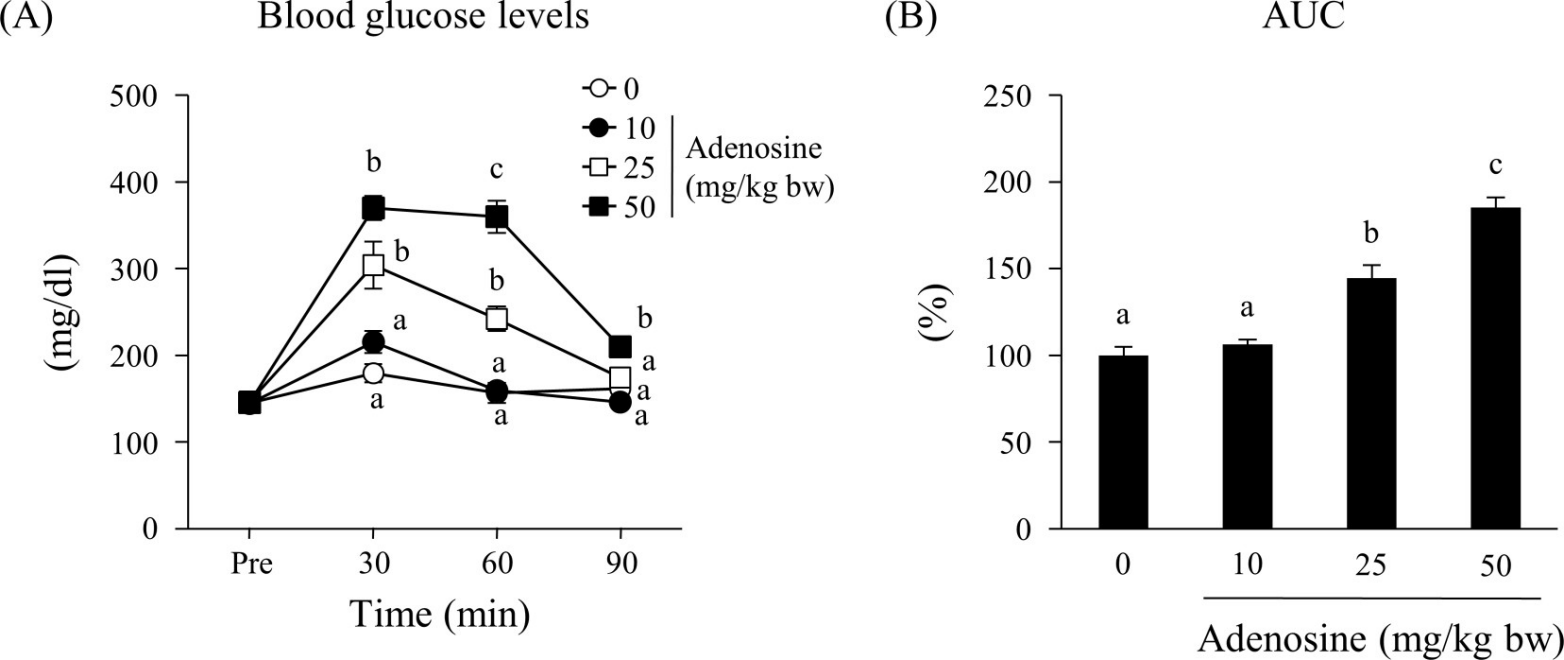
Effect of adenosine on blood glucose levels. (A) Blood was collected from the tail vein just before injection and at 30, 60, and 90 min post subcutaneous injection of 0, 10, 25, or 50 mg/kg bodyweight adenosine. (B) The AUC was calculated from glucose levels during the adenosine injection experiment. Values are presented as the mean ± SEM (n = 6). Groups without a common letter significantly differ (*p* < 0.05).

Fig 1 shows the time course of blood glucose levels and AUC in mice after injecting different doses of adenosine. Until 60 min post injection, adenosine increased blood glucose levels in a dose-dependent manner (Fig 1A). Blood glucose levels in mice administered 25 and 50 mg/kg bw adenosine were significantly higher than those in the other groups. Blood glucose levels peaked 30 min post injection. At 90 min, glucose levels in two adenosine-treated groups (10 and 25 mg/kg bw) decreased to levels similar to those in the control group; however, the highest dose group (50 mg/kg bw) retained a significantly higher glucose level than the other groups. In addition, adenosine dose-dependently increased the AUC (Fig 1B). In the groups injected with 25 and 50 mg/kg bw of adenosine, the AUC increased by 1.4- and 1.9-fold, respectively, compared with the control group. Plasma insulin levels increased at the peak 30 minutes after adenosine injection (S1 Fig). C57BL/6J mice have a spontaneously inactivating Nnt mutation and display glucocorticoid deficiency along with glucose intolerance and reduced insulin secretion. Therefore, the same treatment was performed on C57BL/6N mice without Nnt mutation, and it was confirmed that there was no difference in animal strain (S2 Fig).

### Effect of adenosine on the glycogen content of the liver and skeletal muscle

**Fig 2:**
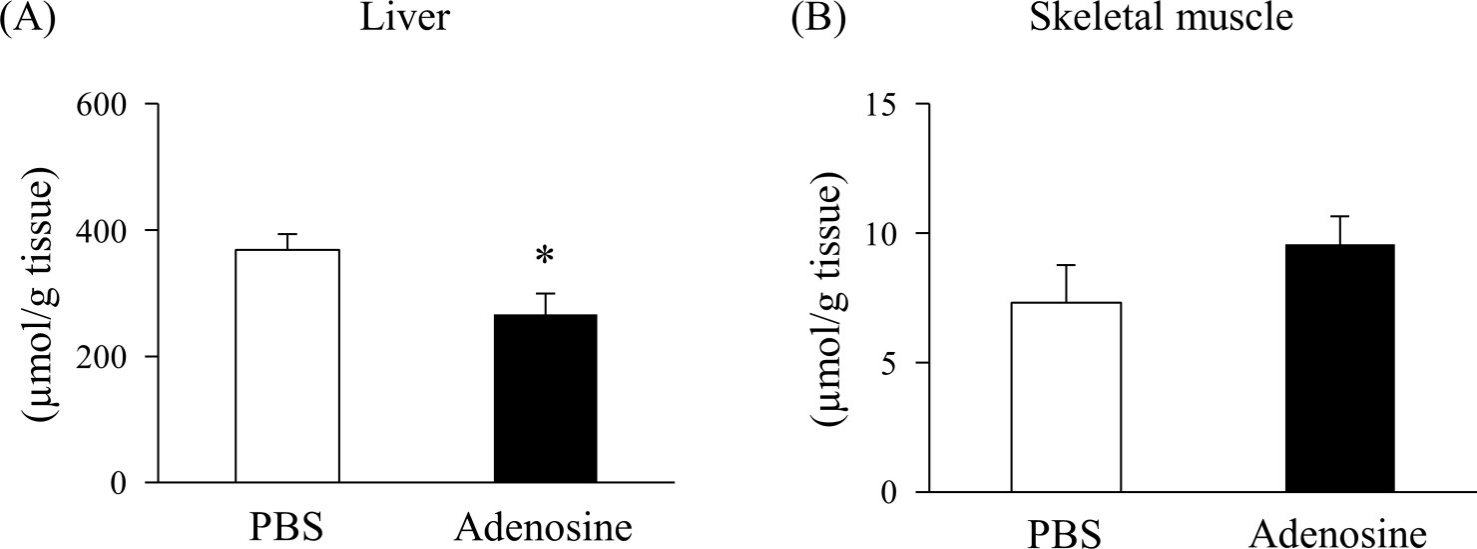
Effect of adenosine on glycogen content. Glycogen contents of the liver (A) and skeletal muscle (quadriceps) (B) were measured at 30 min post subcutaneous injection with PBS or 25 mg/kg bodyweight of adenosine. Values are presented as the mean ± SEM (n = 7–8). * *p* < 0.05 vs. PBS.

The glycogen content of the liver and skeletal muscle in mice 30 min after subcutaneously injecting adenosine (25 mg/kg bw) is displayed in Fig 2. Adenosine significantly decreased glycogen content in the liver compared with the control (Fig 2A). In contrast, no change was observed in the glycogen content of skeletal muscle between the two groups (Fig 2B).

### Effect of adenosine on glycogenolysis after hepatic glycogen depletion

**Fig 3:**
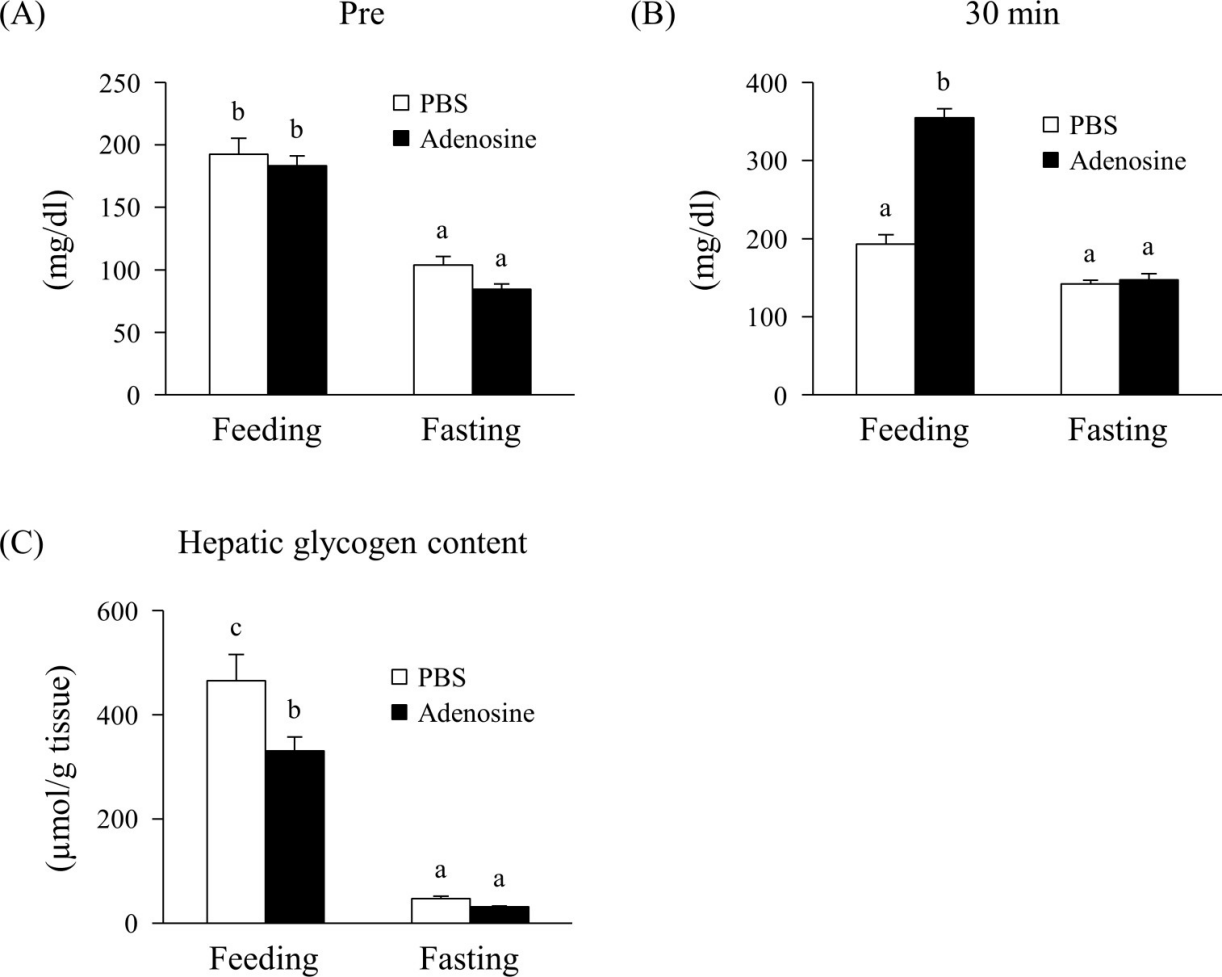
Effect of adenosine on glycogenolysis after hepatic glycogen depletion. Mice were fed *ad libitum* or fasted for 12 h before the experiment. Blood was collected from the tail vein just before injection (A) and at 30 min post subcutaneous injection with PBS or 25 mg/kg bodyweight adenosine (B). Glycogen content of the liver was measured at 30 min post injection (C). Values are presented as the mean ± SEM (n = 5–6). Groups without a common letter significantly differ (*p* < 0.05).

Fig 3 shows the effect of adenosine on glycogenolysis after hepatic glycogen depletion. Blood glucose levels preinjection were significantly decreased by 12 h starvation (Fig 3A). At 30 min after injection, 25 mg/kg bw adenosine significantly increased blood glucose levels (Fig 3B) and decreased hepatic glycogen content in the fed group, but not in the fasting group (Fig 3C). The hepatic glycogen content was significantly decreased by fasting as compared with feeding (Fig 3C).

### Effect of adenosine on the adrenal glands

**Fig 4:**
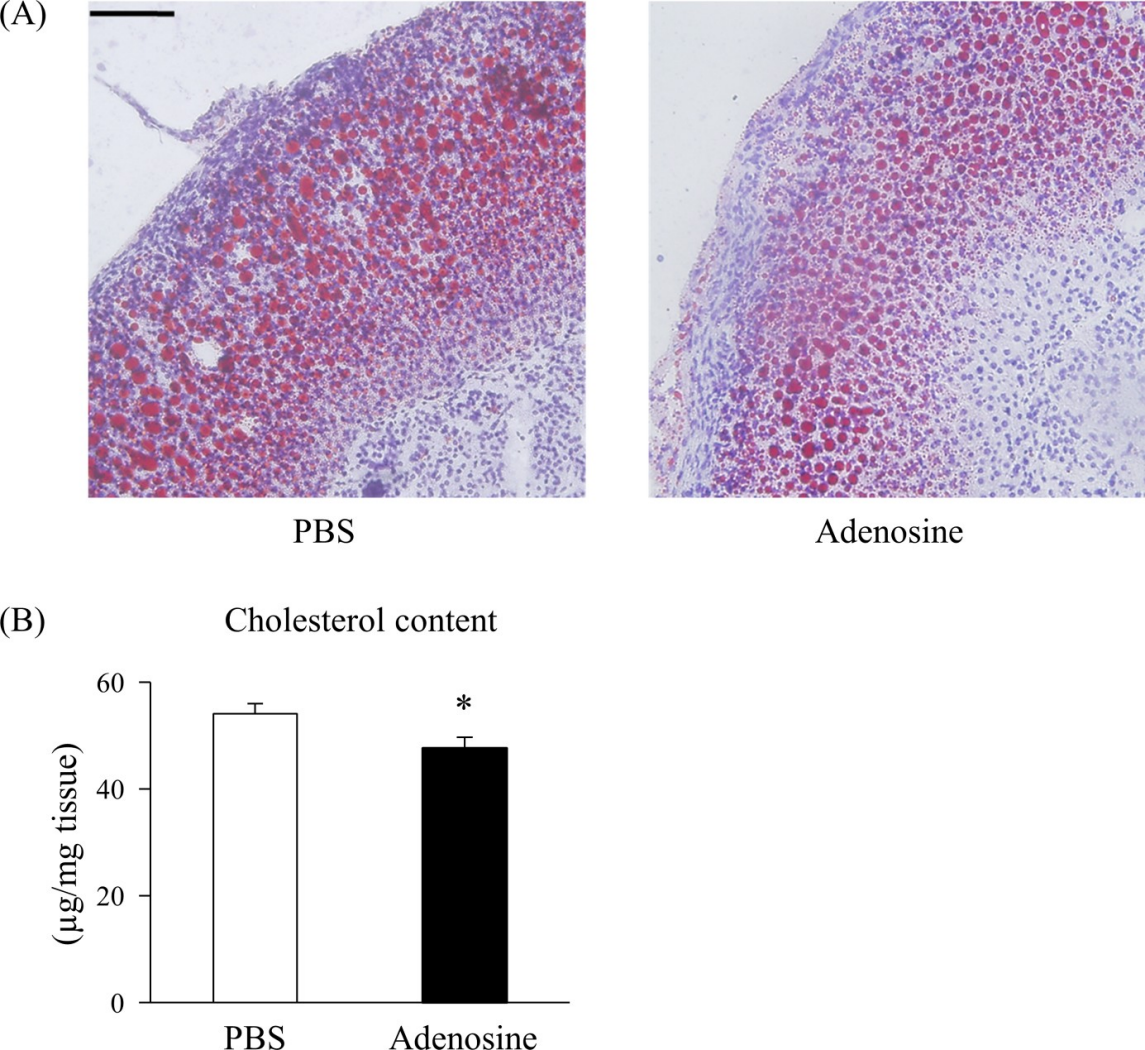
Effect of adenosine on the adrenal glands. (A) Representative Oil red O staining of section from the adrenal glands at 30 min post subcutaneous injection with PBS or 25 mg/kg bw of adenosine. Scale bar: 100 μm. (B) Cholesterol contents of the adrenal glands after the subcutaneous injection. Values are presented as the mean ± SEM (n = 5–6). * *p* < 0.05 vs. PBS.

There was no change in the plasma levels of catecholamine at 30 min post injection (S3 Fig). Representative staining of the adrenal glands with Oil red O at 30 min after 25 mg/kg bw adenosine injection is demonstrated in Fig 4A. The amount of lipid droplets were decreased in the adrenal cortex. The total cholesterol content of the adrenal glands was significantly decreased by adenosine (Fig 4B).

### Effect of adenosine on glycogenolysis signaling in ADX mice

**Fig 5:**
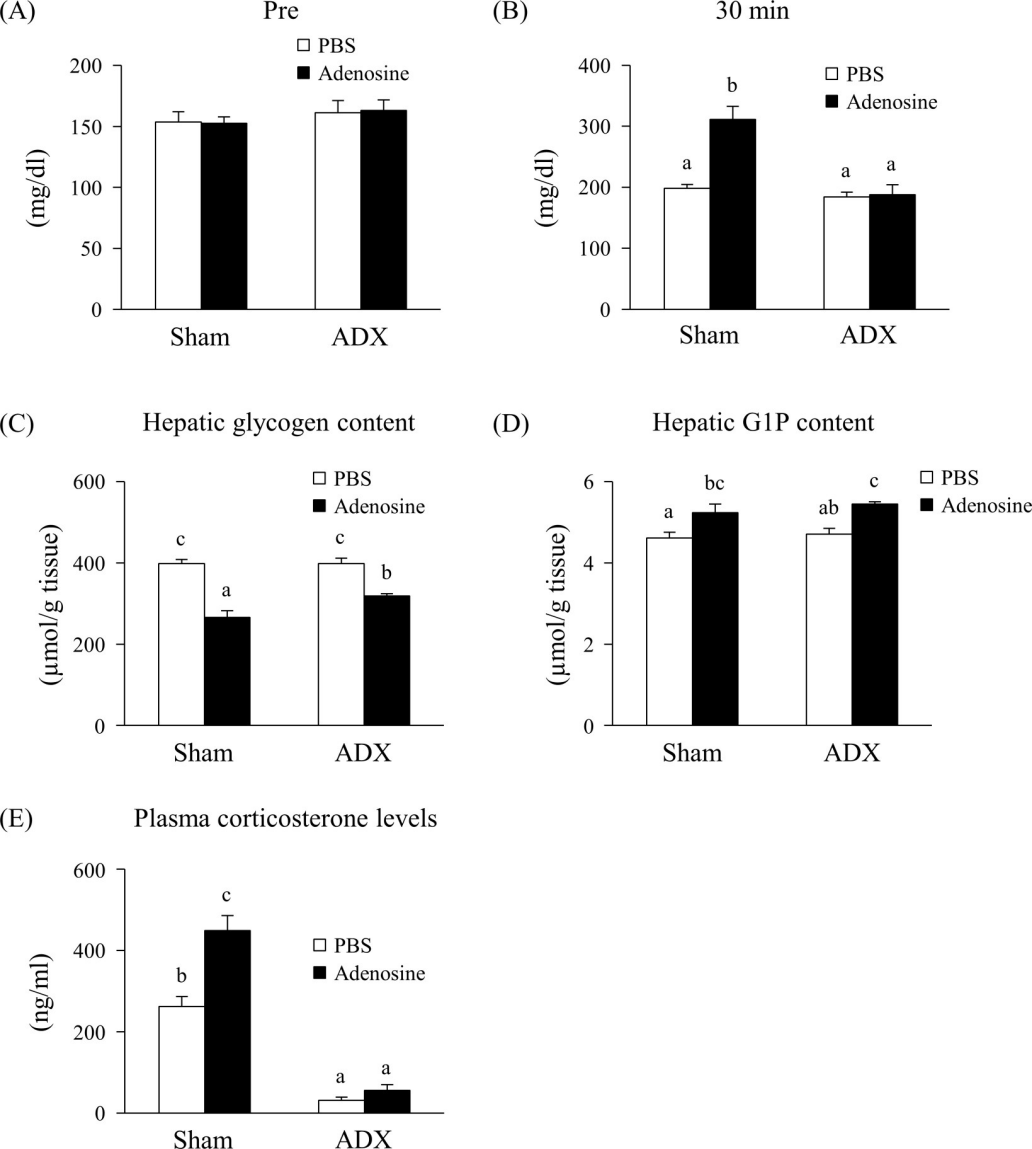
Effect of adenosine on glycogenolysis signaling in ADX mice. Blood was collected from the tail vein just before injection (A) and at 30 min post subcutaneous injection of PBS or 25 mg/kg bw adenosine (B). Glycogen (C) and glucose-1-phosphate (G1P) (D) contents of the liver and plasma corticosterone levels (E) were measured at 30 min post injection. Values are presented as the mean ± SEM (n = 5–6). Groups without a common letter significantly differ (*p* < 0.05).

Fig 5 shows the effect of adenosine on glycogenolysis signaling in ADX mice. No change was observed in the blood glucose levels preinjection (Fig 5A). At 30 min after injection, 25 mg/kg bw adenosine significantly increased the blood glucose levels in the sham operated group, but not in the ADX mice (Fig 5B). Additionally, compared with the sham operated group, the decrease in glycogen content in the liver induced by adenosine was partly suppressed by ADX (Fig 5C). The glucose-1-phosphate, which is metabolite of glycogen and mediated by glycogen phosphorylase, was significantly increased by adenosine injection in both sham operated and ADX operated mice (Fig 5D). Adenosine significantly increased the levels of plasma corticosterone in the sham operated group; however, corticosterone levels were significantly lower in both ADX groups injected with either PBS or adenosine (Fig 5E).

## Discussion

In this study, we examined the effect of adenosine on glucose metabolism *in vivo*. When adenosine was subcutaneously administered to mice, it increased blood glucose levels and the AUC in a dose-dependent manner (Fig 1), suggesting that adenosine enhances glucose production *in vivo*. To clarify the mechanism by which adenosine elevated blood glucose levels, we focused on glycogenolysis. Changes in the content of glycogen were measured 30 min after a subcutaneous injection of 25 mg/kg bw adenosine as this was the minimum dose at which a significant change in the blood glucose level was observed. The significant decrease in the glycogen content in the liver under this condition (Fig 2A) suggests that the enhanced glucose production induced by adenosine results from the degradation of hepatic glycogen. In contrast to the liver, no change in the skeletal muscle glycogen content was observed between the control and adenosine groups (Fig 2B). Although the activation of the A1 adenosine receptor is known to stimulate glucose uptake in skeletal muscle [9, 12], our study showed that adenosine affected neither glycogen breakdown nor synthesis in the muscle at 30 min after injection. To confirm whether the increase in blood glucose levels induced by adenosine depends on hepatic glycogen content, we examined it under fed or fasted conditions. In the fasted state where glycogen was depleted, adenosine had no effect on blood glucose levels and hepatic glycogen content (Fig 3). These results suggest that the increased blood glucose levels induced by adenosine result from the stimulation of hepatic glycogenolysis.

Hepatic glycogenolysis is regulated by adrenal hormones, such as catecholamines and glucocorticoids, *in vivo*. Although there was no change in the levels of plasma catecholamine after adenosine injection (S1 Fig), lipid droplets were decreased in the adrenal cortex (Fig 4A). Furthermore, adenosine injection significantly decreased the level of cholesterol, a precursor of steroid hormone, in the adrenal glands (Fig 4B). These results suggest that adenosine acts on the adrenal glands *in vivo*.

Chen et al. have reported that adenosine promotes steroid hormone synthesis in primary adrenocortical cells is regulated by at least two distinct pathways, the adenosine -A2A/A2B adenosine receptors-JAK2-MEK-ERK cascade and the adenosine- A2A/A2B adenosine receptors-PKCμ-MEK-ERK cascade [13]. Additionally, Chen et al also reported that adenosine increases mRNA expression of steroid synthesis factors, StAR and Cyp11b1, by activation of these A2A/A2B adenosine receptor signals in primary adrenocortical cells. Based on these reports, we analyzed mRNA expression in adrenal gland at post injection of adenosine (S4 Fig). Our data showed that adenosine increased Cyp11b1 mRNA expression in adrenal gland at 10 min after of injection, suggesting adenosine stimulates hepatic glycogenolysis via activation of A2A/A2B adenosine receptor signals and secretion of steroid hormones from adrenal glands in vivo. However, we speculate that the results of mRNA expression at immediately after of injection was also reflect the influence of other factors (handling of mice, injection etc.). In addition, since the effect of adenosine injection on other mechanisms (activity of steroid hormone synthase, extracellular secretion etc.) is not clear, research on the more detailed molecular mechanisms will be required.

To investigate the effect of adenosine on the adrenal gland, we studied glycogenolysis signaling in ADX mice. Adenosine significantly increased blood glucose levels in the sham operated group but not the ADX mice (Fig 5B). Additionally, adenosine decreased the hepatic glycogen content in both groups, but its decrease was smaller in the ADX mice compared with the sham mice (Fig 5C). This result indicates that ADX partially suppresses adenosine-induced hepatic glycogenolysis. The levels of plasma corticosterone biosynthesized from cholesterol in the adrenal cortex were significantly increased in the sham group by adenosine, but its levels were low in ADX groups injected with either PBS or adenosine (Fig 5D). It is known that corticosterone secretion from the adrenal gland is promoted by ACTH secreted from the anterior pituitary gland. Anand-Srivastava et al. have reported that NECA (a non-selective agonist of adenosine receptors) promotes ACTH secretion in cultured anterior pituitary cells [14], but no change in blood ACTH concentration was observed in our experiment (S1 Fig). It has also been reported that adenosine stimulates steroid hormone synthesis via adenosine A2A and A2B receptors in primary adrenal cells, suggesting adenosine acts directly on the adrenal glands *in vivo* [13]. These results suggest that the injection of adenosine promotes secretion of corticosterone from the adrenal cortex, which induces hepatic glycogenolysis and subsequently the elevation of blood glucose levels. However, hepatic glycogenolysis was not completely suppressed in ADX mice (Fig 5C and 5D). The liver produces glucose through glycogenolysis by glycogen phosphorylase, which is activated by cellular cAMP [15]. Previous studies have reported that the stimulation of the perfused liver with adenosine promoted glucose production [16]. Similarly, NECA elevated glycogenolysis in rat primary hepatocytes [10]. Furthermore, activation of the A2b adenosine receptor promoted hepatic glycogenolysis through increasing intracellular cAMP levels *in vitro* [10, 17]. Thus, these reports suggest that adenosine has a direct effect on the liver, and this effect was also demonstrated in ADX mice.

In this study, we have reported that adenosine increased blood glucose levels by stimulating hepatic glycogenolysis. This stimulation is likely to be caused by a direct effect of adenosine on the liver, together with an indirect effect via hormone secretion from the adrenal glands. These findings are useful for clarifying the physiological function of adenosine in glucose metabolism and the mechanism for maintaining blood glucose levels *in vivo*.

## Supporting information

S1 Fig Effect of adenosine on plasma insulin and adrenocorticotropic hormone (ACTH) levels in mice. Blood was collected at post subcutaneous injection with PBS and 25 mg/kg bodyweight adenosine. The levels of insulin and ACTH were determined with kits. Values are presented as the mean ± SEM (n = 5–6).
(TIF)

S2 Fig Effect of adenosine on blood glucose levels in C57BL/6N mice. (A) Blood was collected from the tail vein just before injection and at 30, 60, and 90 min post subcutaneous injection of 0, 25, or 50 mg/kg bodyweight adenosine. (B) The AUC was calculated from glucose levels during the adenosine injection experiment. Values are presented as the mean ± SEM (n = 4).
(TIF)

S3 Fig Effect of adenosine on plasma catecholamine levels in mice. Blood was collected at 30 min post subcutaneous injection with PBS and 25 mg/kg bodyweight adenosine. The levels of adrenalin and noradrenalin were determined with the EIA kits. Values are presented as the mean ± SEM (n = 7).
(TIF)

S4 Fig Effect of adenosine on the expression of genes related to the steroidogenesis in the adrenal glands. The mRNA expression in the adrenal glands were measured at post injection with PBS and 25 mg/kg bw of adenosine. Values are presented as the mean ± SEM (n = 5–6). * *p* < 0.05 vs. PBS.
(TIF)
