# Supplementary figures and images for: Adenosine stimulates hepatic glycogenolysis via adrenal glands–liver crosstalk in mice
Source: PLoS One. 2018 Dec 21;13(12):e0209647. doi: 10.1371/journal.pone.0209647 (PMC6303095; doi:10.1371/journal.pone.0209647)

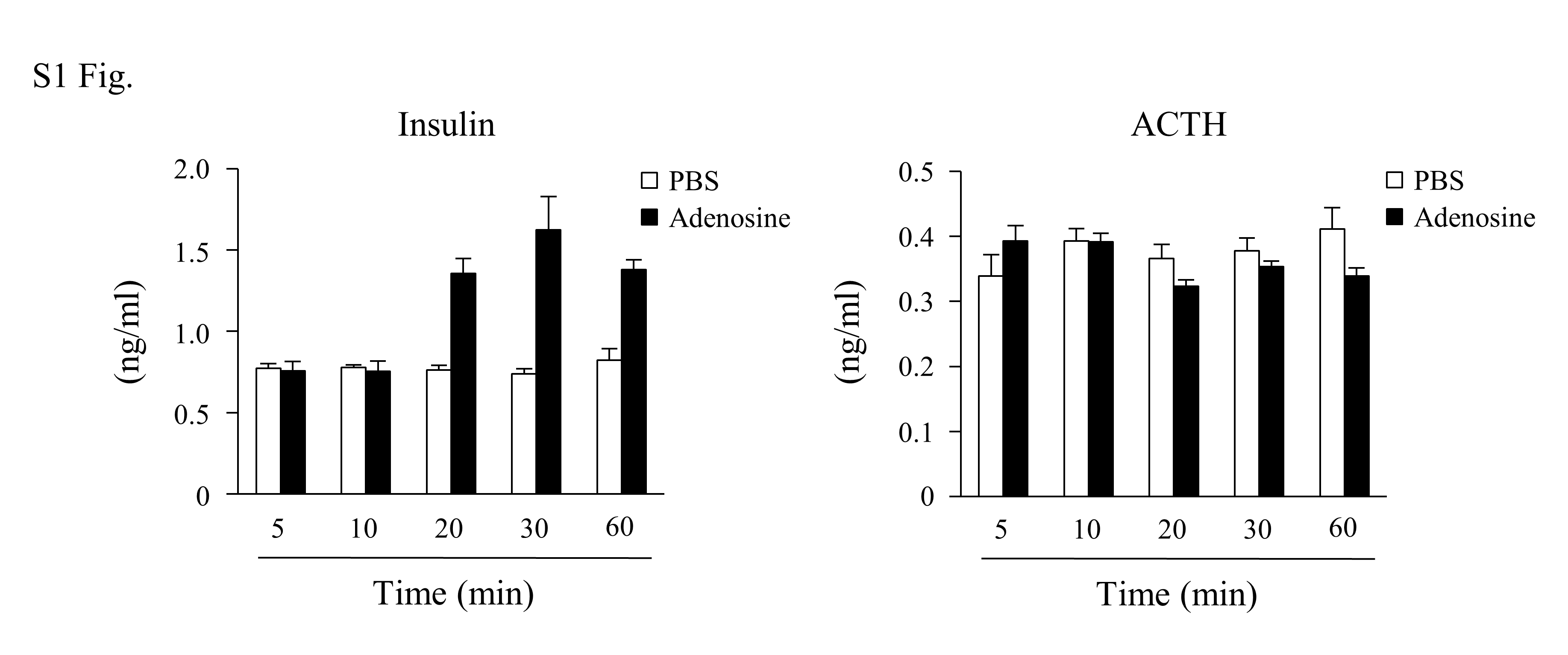

Supplement: S1 Fig — Blood was collected at post subcutaneous injection with PBS and 25 mg/kg bodyweight adenosine. The levels of insulin and ACTH were determined with kits. Values are presented as the mean ± SEM (n = 5–6). (TIF) [file pone.0209647.s001.tif]

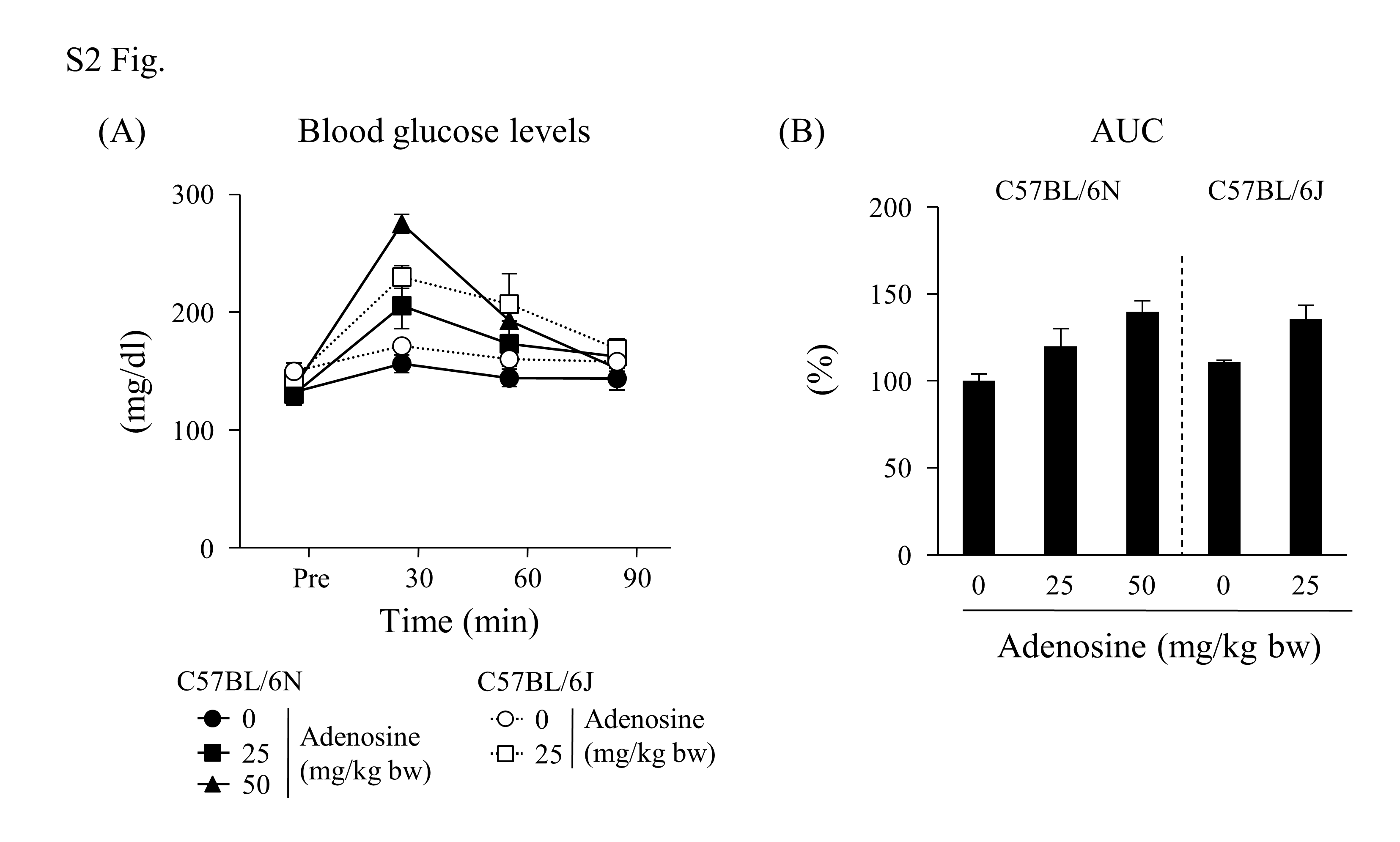

Supplement: S2 Fig — (A) Blood was collected from the tail vein just before injection and at 30, 60, and 90 min post subcutaneous injection of 0, 25, or 50 mg/kg bodyweight adenosine. (B) The AUC was calculated from glucose levels during the adenosine injection experiment. Values are presented as the mean ± SEM (n = 4). (TIF) [file pone.0209647.s002.tif]

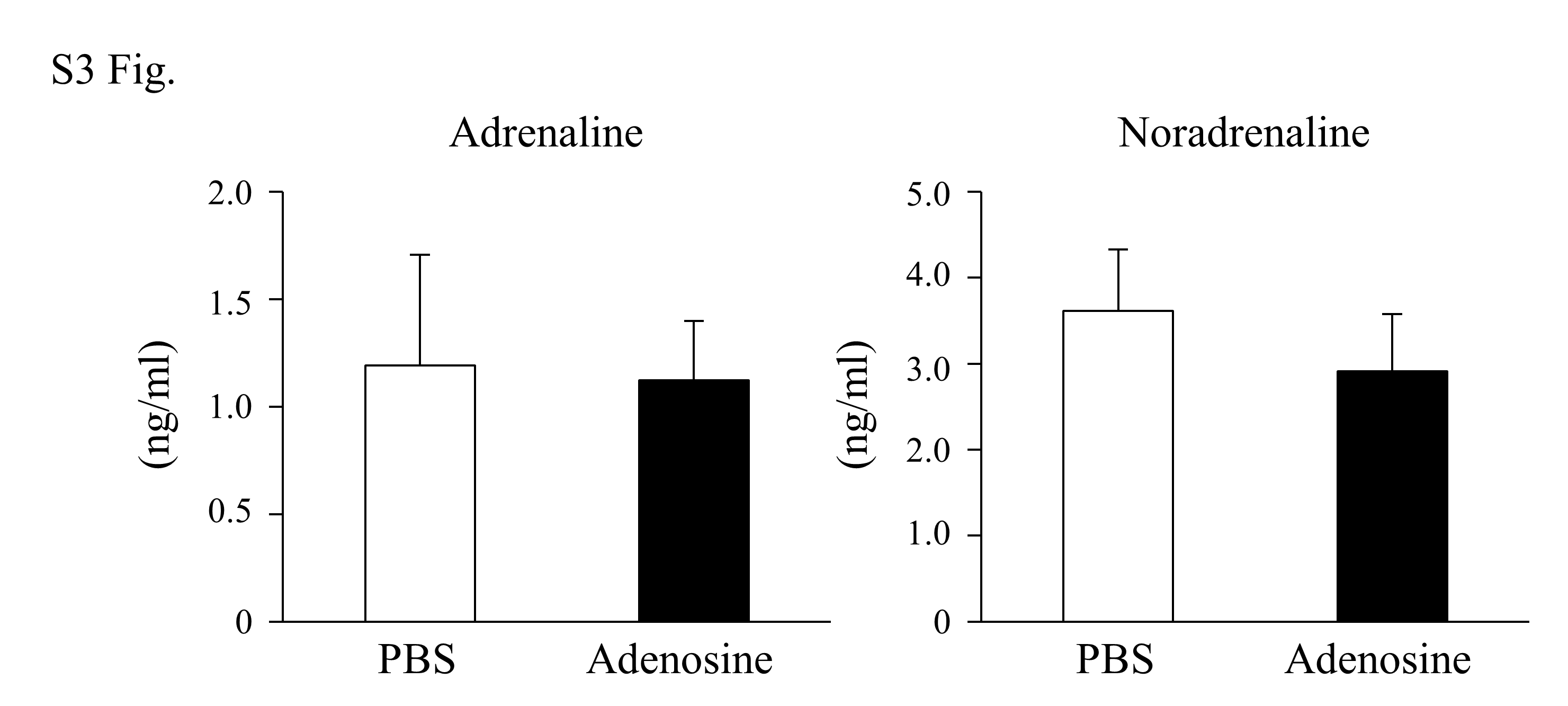

Supplement: S3 Fig — Blood was collected at 30 min post subcutaneous injection with PBS and 25 mg/kg bodyweight adenosine. The levels of adrenalin and noradrenalin were determined with the EIA kits. Values are presented as the mean ± SEM (n = 7). (TIF) [file pone.0209647.s003.tif]

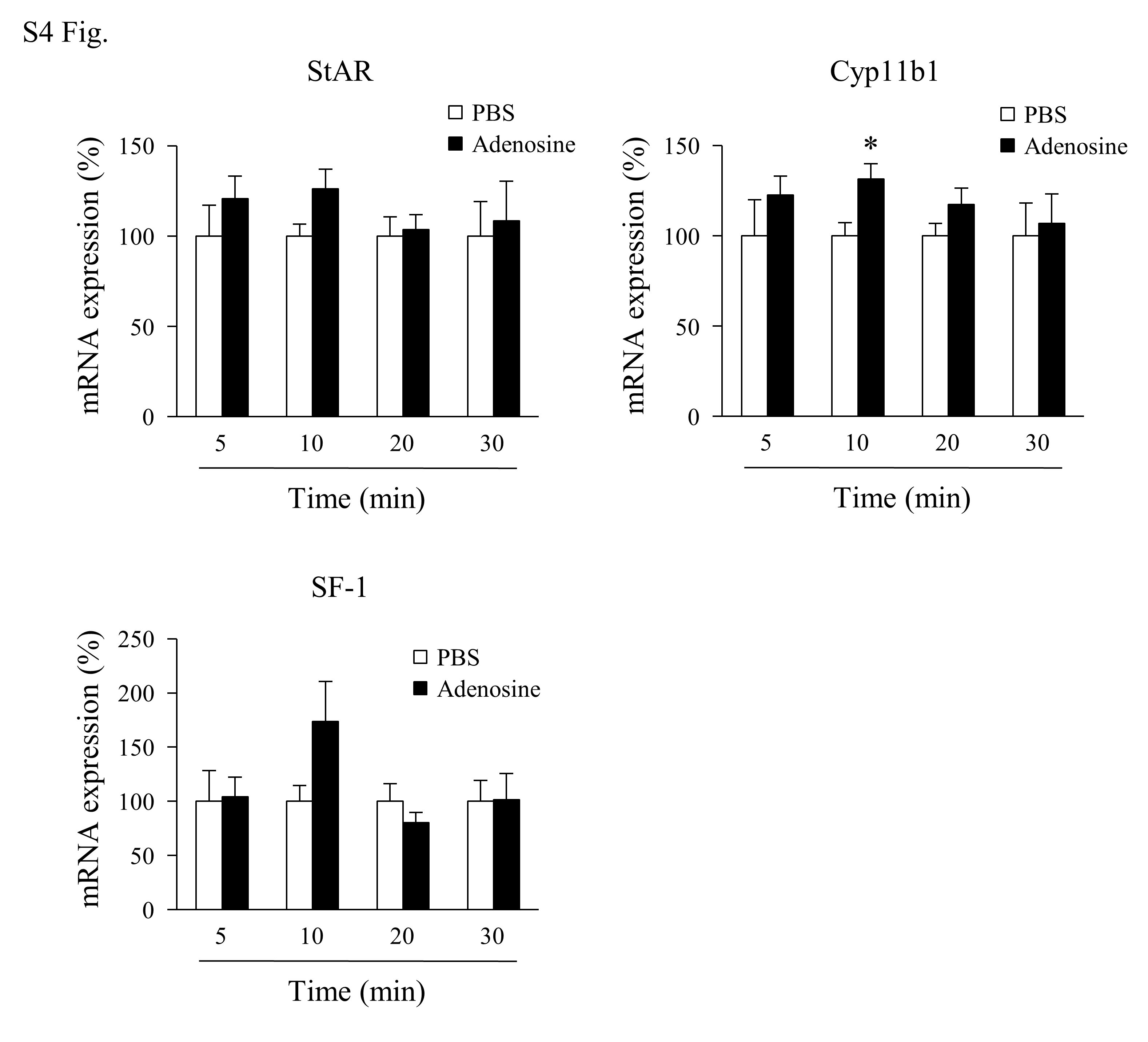

Supplement: S4 Fig — The mRNA expression in the adrenal glands were measured at post injection with PBS and 25 mg/kg bw of adenosine. Values are presented as the mean ± SEM (n = 5–6). * p < 0.05 vs. PBS. (TIF) [file pone.0209647.s004.tif]
